# Cx40 Levels Regulate Hypoxia-Induced Changes in the Migration, Proliferation, and Formation of Gap Junction Plaques in an Extravillous Trophoblast Cell Model

**DOI:** 10.3390/cells13131150

**Published:** 2024-07-06

**Authors:** Fernanda M. Rozas-Villanueva, Viviana P. Orellana, Rodrigo Alarcón, Jaime Maripillan, Agustin D. Martinez, Ivan E. Alfaro, Mauricio A. Retamal

**Affiliations:** 1 Programa de Comunicación Celular en Cáncer, Facultad de Medicina Clínica Alemana, Universidad del Desarrollo, Santiago 7550000, Chile; fernanda.rozas.v@gmail.com (F.M.R.-V.); tm.vorellana@gmail.com (V.P.O.); rodalj@gmail.com (R.A.); ialfaro@udd.cl (I.E.A.); 2 Centro Interdisciplinario de Neurociencias de Valparaíso, Instituto de Neurociencia, Facultad de Ciencias, Universidad de Valparaíso, Valparaíso 2360102, Chile; jaime.maripillan@gmail.com (J.M.); agustin.martinez@uv.cl (A.D.M.); 3 Center for Membrane Protein Research, Department of Cell Physiology and Molecular Biophysics, Texas Tech University Health Sciences Center, Lubbock, TX 79430, USA

**Keywords:** connexins, extravillous trophoblast, gap junction channels, placenta, nitric oxide

## Abstract

Background: Extravillous trophoblasts (EVTs) form stratified columns at the placenta–uterus interface. In the closest part to fetal structures, EVTs have a proliferative phenotype, whereas in the closest part to maternal structures, they present a migratory phenotype. During the placentation process, Connexin 40 (Cx40) participates in both the proliferation and migration of EVTs, which occurs under hypoxia. However, a possible interaction between hypoxia and Cx40 has not yet been established. Methods: We developed two cellular models, one with “low Cx40” (Jeg-3), which reflected the expression of this protein found in migratory EVTs, and one with “high Cx40” (Jeg-3/hCx40), which reflected the expression of this protein in proliferative cells. We analyzed the migration and proliferation of these cells under normoxic and hypoxic conditions for 24 h. Jeg-3 cells under hypoxia increased their migratory capacity over their proliferative capacity. However, in Jeg-3/hCx40, the opposite effect was induced. On the other hand, hypoxia promoted gap junction (GJ) plaque formation between neighboring Jeg-3 cells. Similarly, the activation of a nitro oxide (NO)/cGMP/PKG-dependent pathway induced an increase in GJ-plaque formation in Jeg-3 cells. Conclusions: The expression patterns of Cx40 play a crucial role in shaping the responses of EVTs to hypoxia, thereby influencing their migratory or proliferative phenotype. Simultaneously, hypoxia triggers an increase in Cx40 gap junction (GJ) plaque formation through a pathway dependent on NO.

## 1. Introduction

The main functions of the placenta include facilitating the exchange of waste metabolites (such as CO_2_), molecular oxygen (O_2_), and nutrients between the fetus and the maternal circulatory system. The placenta is composed of various cell types, with cytotrophoblasts and syncytiotrophoblasts forming the floating villi and anchoring villi, respectively [1]. Another crucial cell type is extravillous trophoblasts (EVTs), which organize into stratified structures resembling columns at the placenta–uterus interface [1]. In the proximal part of these structures, EVTs exhibit a high self-renewal capacity [2]. However, this characteristic diminishes progressively towards the distal part of the column, and it is replaced by the acquisition of a mesenchymal phenotype. This transformation enables EVTs to exit the columns and penetrate maternal structures, ultimately participating in the remodeling of spiral arteries [3,4,5]. Therefore, the processes of migration and invasion play pivotal roles in the final stages of blood communication between the fetus and its mother. Failure in this remodeling process can lead to pathological conditions such as preeclampsia [6].

Until the 12th week of gestation, the placentation process unfolds within a hypoxic environment (1–2% O_2_) [7,8,9,10]. Intriguingly, there is a consensus that low O_2_ levels enhance the migratory characteristics of extravillous trophoblasts (EVTs). For instance, Genbacev et al. observed that first-trimester placental explants, when grown under hypoxic conditions, exhibited prominent cell columns with cells displaying heightened migration compared to explants grown under standard oxygen conditions (21%) [10,11,12]. It is well-established that hypoxia primarily exerts its effects through the activation of hypoxia-inducible factors (HIFs) [13,14]. However, paradoxically, the activation of HIF-1α reduces EVTs’ migratory capacity [15,16,17], suggesting that the overall impact of hypoxia on EVTs is intricate and involves pathways beyond HIF activation.

The placental process demands precise regulation to achieve optimal cell differentiation and migration. This regulation is, in part, orchestrated by autocrine and paracrine communication among neighboring cells. Among the proteins facilitating intercellular communication, connexins (Cxs) play a vital role. The assembly of six Cxs forms a hemichannel, which can either embed itself in the plasma membrane as a free hemichannel or dock with another hemichannel from a neighboring cell to create a gap junction channel (GJC). Hemichannels and GJCs permit the direct passage of ions and small molecules [18,19,20], limited by a pore diameter typically ranging from 8 to 15 Å for certain Cxs [21,22,23].

In the human placenta, only five members of the Cxs family have been identified thus far, and among them, only Cx40 and Cx43 have been attributed specific functions [24]. Cx43 is found in syncytiotrophoblasts of floating villi, and its presence is associated with the fusion of cytotrophoblasts to form syncytiotrophoblasts [24]. This relationship is evident from studies utilizing Cx43 anti-sense, which results in a reduction in syncytiotrophoblast formation [25,26]. Conversely, Cx40 has undergone extensive study and is now established to be present in extravillous trophoblast (EVT) cells [27,28,29]. Its presence is correlated with both an increase in the expression of the proliferative marker Ki67 and a decrease in motility [28]. This implies an inverse relationship between the expression of Cx40 and/or its function as a GJC and the migration capacity of EVTs.

As presented, hypoxia and Cx40 modulate EVTs’ migration and proliferation. Nevertheless, there has been no exploration of whether these two conditions might interact with each other. Therefore, our primary objective was to investigate a potential relationship between the expression levels of Cx40 and the response to hypoxia in EVT cells.

## 2. Materials and Methods

### 2.1. Cell Culture

The Jeg-3 cell line, derived from choriocarcinoma, has been extensively employed as a model for trophoblast studies [30]. This cell line was provided by Dr. Jaime Gutiérrez of Universidad San Sebastián at Santiago, Chile. Both Jeg-3 and Jeg-3/hCx40 cell lines were cultured in RPMI supplemented with 10% fetal bovine serum (FBS), streptomycin (100 μg), and penicillin (100U) (P/S). Additionally, HeLa wild type (WT) and HeLa cells expressing human Cx40 (hCx40) were cultured in DMEM supplemented with 10% FBS, streptomycin (100 μg), and penicillin (100U) (P/S). All cells were maintained under standard conditions at 37 °C, 5% CO_2_, and 95% humidity.

### 2.2. Overexpression of Cx40

The expression plasmid pLV [Exp] -EGFP: T2A: Puro-EF1A-hGJA5 was obtained from Vector Builder. This plasmid incorporates the coding sequence for the protein “Gap junction alpha 5” or hCx40, along with enhanced green fluorescent protein (EGFP) and a Puromycin (Puro) resistance gene. The latter facilitated the selection of transfected cells. Jeg-3 or HeLa cells in culture were transfected using Lipofectamine 3000 in accordance with the manufacturer’s instructions (Thermo Fisher Scientific, Waltham, MA, USA). Subsequently, the transfected cells were subjected to selection for 14 days using Puromycin at a concentration of 1 µg/mL.

### 2.3. Western Blot

The cells were seeded in 60 mm plates, and when they reached 80% confluence, they were washed with phosphate buffer saline (PBS) (in mM: 137 NaCl, 2.7 KCl, 10 Na_2_HPO_4_, 1.8 KH_2_PO_4_, pH: 7.4). Then, 500 mL of RIPA buffer (in mM: 150 NaCl, 20 Tris pH: 7.4, 0.5% Sodium Desoxycholate, 0.1% Sodium Dodecyl Sulfate (SDS), 1% Nonidet P-40, and a protease inhibitor cocktail tablet) was added, and the cells were immediately harvested with a cell scraper, sonicated, and centrifuged at 4° C and 13,500× *g* for 2 min. The supernatant was then taken and protein quantification was performed using the Qubit protein Assay Kit on a Qubit Fluorometer (Thermo Fisher Scientific, Waltham, MA, USA) following the manufacturer’s instructions. Subsequently, the proteins were denaturated by adding Laemli buffer (62.5 mM Tris pH 6.8, 25% glycerol, 2% SDS, 0.01% bromophenol blue, and 5% β-mercaptoethanol) and heated at 95 °C for 3 min. Protein samples were placed in a 10% polyacrylamide gel and then transferred to a polyvinylidene difluoride (PVDF) membrane by cold wet transfer for 2 h at 100 V in Tris-buffer, glycine 20% methanol. The PVDF membrane was blocked with 5% non-fat milk in 0.05% T-TBS (TBS and 0.05% Tween 20) for 1 h at room temperature (RT) with stirring. After blocking, the membrane was incubated overnight at 4 °C in shaking primary antibody (Rabbit (Rb) anti-Cx40, or Mouse (Ms) anti-Tubulin, both Invitrogen, Waltham, MA, USA). Subsequently, the membrane was washed 3 times with 0.05% T-TBS for 10 min each and incubated with the corresponding secondary antibody conjugated with peroxidase (HRP) for 1 h while shaking at RT (Goat anti-Rb-HRP, Invitrogen, Waltham, MA, USA, or Rabbit anti-Ms-HRP, Abcam, Cambridge, UK). The membranes were revealed by chemiluminescence using a C-Digit Blot Scanner (Li-Cor Biosciences, Lincoln, NE, USA).

### 2.4. Immunofluorescence

The cell lines were seeded in 12 mm glass coverslips. After 48 h, having been exposed for 24 h to the different experimental conditions, the covers were washed with PBS and fixed/permeabilized with methanol–acetone (1:1) at −20 °C for 20 min. Then, they were washed with PBS 3 times, and the covers were blocked with a 5% BSA solution in PBS for 2 h at RT in a humid chamber. The primary antibody (Rb anti-Cx40 Invitrogen, Waltham, MA, USA, or Rb anti-Hif-1α) was diluted in 1% BSA in PBS and the covers were incubated overnight at 4° C. The excess antibody was washed 3 times with PBS and the secondary antibody (conjugated to Alexa 594) diluted in 1% BSA in PBS was added and incubated for 2 h at RT. After that time, they were washed again 3 times with PBS and the covers were mounted on a slide with a drop of DAPI-Fluoromont. Photographs were taken under an inverted fluorescence microscope (Nikon model Eclipse Ti, Tokio, Japan) or at 60X magnification on a Leica TCS SP8 MP confocal microscope. The images were analyzed using ImageJ software (Version 1.53q). Note: we opted to modify the color of the secondary antibody signal from red to green. This adjustment aimed to enhance the visual contrast against the background, as well as the blue signal emitted by DAPI staining.

### 2.5. Hypoxic Condition

The cells were cultured on 12 mm glass coverslips. On the day of the experiment, the cells underwent three washes with saline media comprising the following components (in mM): 140 NaCl, 4 KCl, 2 CaCl_2_, 1 MgCl_2_, 5 glucose, and 10 HEPES, adjusted to a pH of 7.4. Subsequently, the cells were immersed in the same saline medium, supplemented with 10% FBS. Following this, the cells were enclosed within a hypoxia chamber (Billups-Rothenberg Inc., San Diego, CA, USA), featuring a 60 mm plastic plate filled with autoclaved water to maintain the humidity levels. The chamber was sealed, and the ambient air was exchanged with a 100% nitrogen gas flow for 5 min, succeeded by the injection of a gas mixture containing 1% oxygen and 99% nitrogen for an additional 5 min. After preparing the chamber, it was placed in an oven set at 37 °C for 24 h. To confirm the success of the experiment, an immunofluorescence assay for HIF-1a was conducted.

### 2.6. Activation of the NO-cGMP-PKG Pathway

To assess the impact of this pathway, the cells underwent treatment with specific compounds: 1 mM Sodium Nitroprusside (SNP) (Sigma-Aldrich, St Louis, MO, USA), 500 µM Diethylamide NONOate (Cayman Chemical), 500 µM S-nitrosoglutathione (GSNO) (Cayman Chemical, Ann Arbor, MO, USA), or 1 mM 8-bromo-cGMP (Tocris Bioscience, Bristol, UK)—a cGMP analog known for its increased stability, resistance to phosphodiesterase (PDE) activity, and membrane permeability. Additionally, a protein kinase inhibitor, KT5823 (Tocris Bioscience, Bristol, UK), was utilized at concentrations of 1 µM as an inhibitor of protein kinase G (PKGi). Furthermore, a Nitric Oxide synthetase inhibitor, 1 mM L-name (Sigma-Aldrich, St Louis, MO, USA), was employed. All treatments were extended for 24 h, except for treatments involving PKGi, which commenced 2 h before cGMP addition. Given that the reagents (excluding SNP) were dissolved in Dimethyl sulfoxide (DMSO), control experiments with DMSO as the vehicle were incorporated into these assays.

### 2.7. Cell Death Assays

For the MTS assay, the cells were initially seeded at 5 × 10^3^ cells per well in 96-well plates with RPMI and 10% FBS, followed by 24 h of incubation. Subsequently, the cells were treated with fresh RPMI containing 0.5 mM or 1 mM of Sodium Nitroprusside (SNP) and further incubated for 24 h at 37 °C, 5% CO_2_, and 95% humidity. Cell viability was assessed using the CellTiter 96 Aqueous Non-Radioactive Cell Proliferation Assay (Promega, Madison, WI, USA), measuring the amount of colored formazan dye produced. The plates were read at an absorbance of 490 nm one to two hours after adding the MTS reagent using a microplate reader (BioTek, Cytation3, Santa Clara, CA, USA). For a 7-AAD uptake analysis, the cells were initially grown on 12 mm glass coverslips until reaching approximately 80% confluence. The culture medium was then replaced with saline media supplemented with 10% FBS, and the cells were exposed to either 500 µM Diethylamide NONOate (NONOate), 500 µM S-nitrosoglutathione (GSNO), or 1 mM SNP for 24 h. Following this incubation, the culture medium was replaced with a fresh saline medium containing 10 μM 7-AAD. After 10 min of incubation at room temperature, the cells underwent three washes and images were captured using a 20X magnification epifluorescent microscope (Nikon, Ti-U). As a positive control, a coverslip with cells was exposed to 0.5% Triton X-100 for 5 min, washed three times with saline medium, and then exposed to 7-AAD.

### 2.8. Scrape Loading Assay

Gap junction permeability was evaluated at room temperature utilizing the scrape-loading/dye transfer technique. For this experiment, Jeg-3 cells were cultured at 90–100% confluence, subjected to three washes with a divalent cation-free solution (DCFS) (in mM: 140 NaCl, 4 KCl, 5 glucose, and 10 HEPES, pH 7.4), and then incubated with DCFS supplemented with DAPI (50 µM). Subsequently, a controlled scrape-loading procedure was performed using a razor blade. Following 20 min of incubation, the culture plates were gently rinsed three times with saline media (see hypoxic condition) without DAPI and examined under epifluorescence microscopy (Nikon model Eclipse Ti, Tokio, Japan).

### 2.9. Cell Proliferation Assay

In 35 mm culture plates, 20,000 cells were seeded per plate and cell counting was performed by the means of trypan blue exclusion at times of 0, 24, 48, and 72 h. For this, the plates were washed with PBS and then added to 300 µL of trypsin-EDTA (0.05%). Then, they were incubated for 5 min at 37 °C and 300 µL of complete medium was added. With the aid of a pipette, the medium was homogenized and the cells were dispersed. Of the 600 µL total, a 15 µL aliquot was taken, mixed 1: 1 with trypan blue, and homogenized with the pipette. Of this solution, 10 µL was taken to load into each of the compartments of a Neubauer chamber. Therefore, each time was counted in duplicate. This test was repeated 3 times independently.

### 2.10. Cell Migration Assay

In 35 mm culture plates, 200,000 cells were seeded per plate to establish a cell monolayer within 48 h. Once the monolayer was formed, a straight-line wound was created using a yellow micropipette tip. Subsequently, the cells underwent two PBS washes to eliminate cell debris, followed by the addition of complete medium. The cells were then incubated at 37 °C and photographs were captured at 0, 2, 4, 8, 10, and 24 h. In the case of hypoxia tests, only the time points of 0 and 24 h were documented due to the constraints posed by the test conditions, preventing the cells from being re-oxygenated at each time interval.

### 2.11. Statistical Analysis

Data are shown as averages with their respective standard errors (SEM) for continuous variables, while for categorical variables and wound healing tests, they are shown as percentages. The statistical differences of continuous variables between two groups were evaluated with Student’s *t*-test, while if a comparison was made with more than two groups, ANOVA was used. Values less than 5% (*p* < 0.05) were considered as significant *p*. The data were analyzed using Prisma 9.0 software (GraphPad Software Inc, La Jolla, CA, USA) and STATA 16 software (StataCorp, College Station, Tx, USA).

## 3. Results

### 3.1. Hypoxia Induces Cx40 GJ Plaques Formation between Jeg-3 Cells

**Figure 1:**
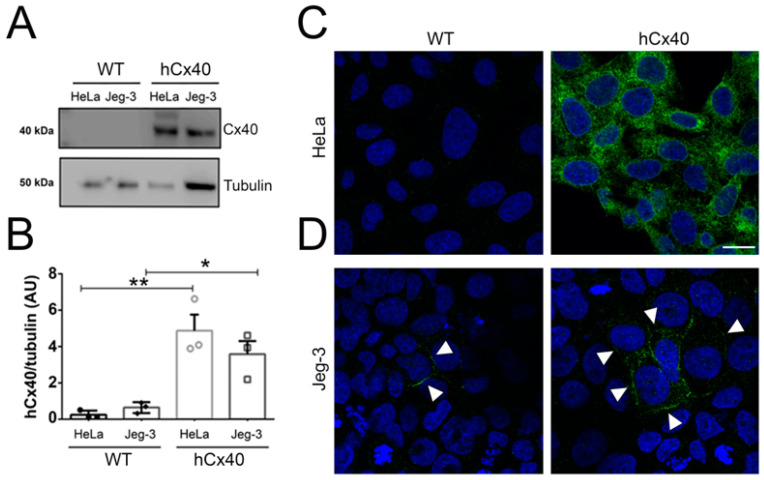
Immunodetection of Cx40. (**A**) Evaluation of the Cx40 protein through Western Blot analysis using Jeg-3 and Hela cells, both transfected and non-transfected with hCx40. HeLa cells were employed as an expression control and for assessing the specificity of the primary antibody. Each lane was loaded with 60 µg of total proteins. The upper panel shows the results of the primary anti-Cx40 antibody, while the lower panel displays anti-tubulin, with both revealed using a secondary antibody conjugated to HRP. (**B**) Densitometry quantification of the bands shown in (**A**). Statistical comparisons were made using the t-test against the respective controls, with significance indicated as * *p* < 0.05 and ** *p* < 0.01. Error bars represent the standard error of n = 3. Detection of Cx40 through indirect immunofluorescence, recorded using confocal microscopy. (**C**) The upper panels illustrate the localization of Cx40 in HeLa cells, both wild type (WT) and those transfected with Cx40 (hCx40). (**D**) The lower panels show Jeg-3 cells, both WT and hCx40. Arrow heads show localization of Cx40 GJ plaques. Scale bar = 10 mm. A secondary anti-rabbit antibody used in this study was conjugated with Alexa Fluor 594 (signal showed in green), while nuclei were stained with DAPI.

As previously mentioned, EVTs can be stratified into two groups based on their levels of Cx40 expression, a characteristic that correlates with their proliferative and migratory capabilities [27,28]. In our experiments, the Jeg-3 cells exhibited nearly undetectable levels of Cx40 expression when assessed through Western blot analysis (Figure 1A). However, immunoreactive signals were obvious using confocal microscopy (Figure 1D, JEG-3 WT). In this context, the Cx40 signal was faint and observed in only a few cells, where it localized within the cytoplasm and, in some instances, at cell-to-cell contacts forming gap junction (GJ) plaques (Figure 1D, WT). Conversely, Jeg-3 cells stably overexpressing Cx40 (Jeg-3/hCx40 cells) exhibited clear Cx40 immunodetection in both the Western blot and immunofluorescence analyses (Figure 1A,D, hCx40). In this case, the Cx40 signaling was substantially more pronounced compared to that in the Jeg-3 cells, and the presence of GJ plaques was frequently observed (Figure 1D, hCx40). These results suggest that the antibody used in this study demonstrates a considerably higher sensitivity to Cx40 in immunofluorescence than in Western blot analyses. To confirm the antibody’s specificity, HeLa cells, known to have very low, if any, endogenous expression of Cxs, were employed. Western blot analysis revealed that non-transfected HeLa cells (HeLa WT) did not exhibit detectable levels of endogenous Cx40. In contrast, HeLa cells overexpressing Cx40 displayed two immunoreactive bands, with one corresponding to the expected theoretical weight of approximately 40 kDa (Figure 1A, hCx40). Upon normalization by tubulin expression, the expression differences between the transfected cells and their parental counterparts were found to be statistically significant (Figure 1B). Interestingly, HeLa hCx40 immunofluorescence was predominantly found in the cytoplasm, with no apparent GJ plaques (Figure 1C, hCx40). Therefore, we established a cellular model encompassing both low and high Cx40 expression levels, resulting in distinct patterns of GJ plaque formation.

**Figure 2:**
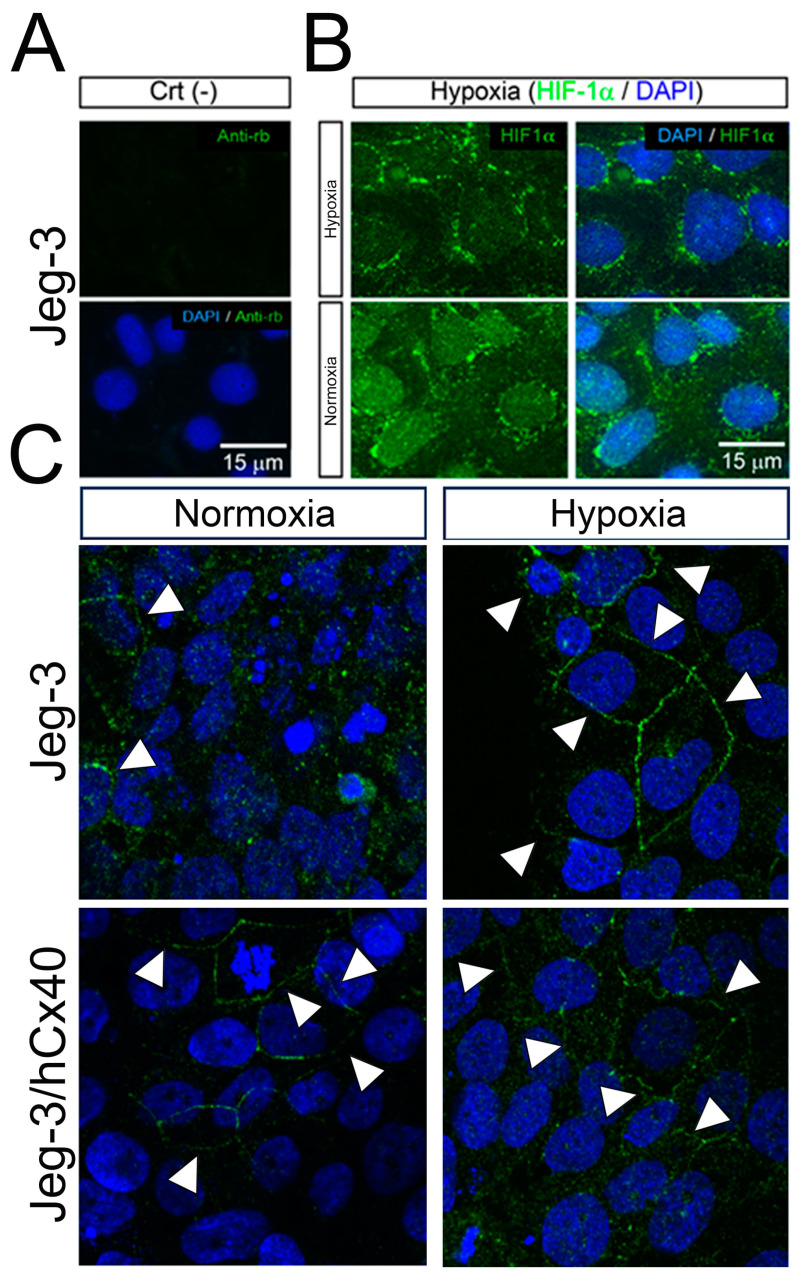
Hypoxia induces Cx40 GJ plaque formation in Jeg-3 cells. Jeg-3 and Jeg-3/hCx40 cells were grown in normoxic and hypoxic conditions for 24 h. (**A**) Immunofluorescence negative control performed using cells incubated only with secondary anti-Rabbit antibody conjugated with FITC. (**B**) Immunodetection of Hif-1α (green signal) in Jeg-3 cells: In normoxia, the immunoreactive signal for Hif-1α was observed mostly in perinuclear regions. However, under hypoxia, Hif-1α was translocated to the nucleus. (**C**) Immunodetection of Cx40 (green signal): left panels cells maintained in normoxia and right panels cells maintained in hypoxia for 24 h. Upper panels correspond to Jeg-3 cells, and lower panels correspond to Jeg-3/hCx40 cells. Representative photographs of 3 independent experiments. Arrow heads show Cx40 GJ-plaques and scale bar = 15 µm.

We then investigated whether hypoxia could influence the cellular localization of Cx40. To validate the efficacy of our hypoxia protocol, the translocation of Hif-1α from the cytoplasm to the nucleus was assessed through immunofluorescence. In our negative control, where no primary antibody was added, no signals were detected (Figure 2A, Crt (-)). Under normoxic conditions, Hif-1α predominantly resided in perinuclear regions, but after 24 h of hypoxia, it translocated to the cell nucleus (Figure 2B). Regarding Cx40 in normoxic conditions, it was primarily located in the cytoplasm of Jeg-3 cells, with some instances of localization at cell-to-cell contacts forming faint GJ plaques (Figure 2C, white arrow). However, following 24 h of hypoxia exposure, there was a noticeable increase in both the quantity and thickness of the GJ plaques (Figure 2C, white arrow). In the case of Jeg-3/hCx40 cells, GJ plaques were observed under both normoxic and hypoxic conditions (Figure 2C, lower panels). Our findings suggest that hypoxia promotes the formation of Cx40-associated GJ plaques in Jeg-3 cells with “low” levels of Cx40, but it does not have a significant effect on cells with higher expression levels.

### 3.2. GJCs Induced by Hypoxia Are Functional

**Figure 3:**
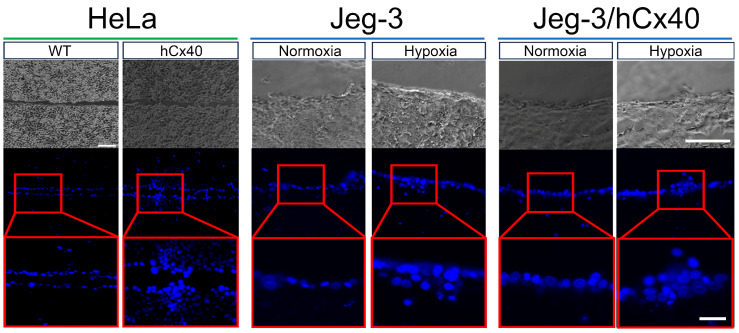
Hypoxia induces an enhanced DAPI transfer between neighboring cells. To assess intercellular coupling in 90–100% confluent cell cultures, a DAPI (50 μM) scrape loading assay was conducted. Upper panels correspond to the phase-contrast images for the lower panels (Scale bar = 100 mm). The middle panels display images captured at a 20X magnification using a 350 nm filter in an epifluorescence microscope. The lower panels offer a closer view of the highlighted red areas in the middle panels. Each image is a representative sample from a total of n = 3 independent experiments. Scale bar = 30 µm.

As demonstrated earlier, hypoxia induced an increase in GJ plaques, suggesting heightened intercellular communication. To investigate this possibility further, we conducted a dye transfer scrape-loading assay (Figure 3). As expected, HeLa WT cells exhibited no DAPI transfer between adjacent cells (Figure 3, left panel, WT). In contrast, HeLa hCx40 cells displayed clear DAPI transfer between cells, extending even to those distant from the initial scratch (Figure 3, left panel). Under normoxic conditions, Jeg-3 cells remained uncoupled, aligning with their low degree of GJ plaques between cells (see Figure 2). However, in hypoxic conditions, Jeg-3 cells exhibited patches of DAPI transfer between cells (Figure 3, center panel), consistent with the patched presence of Cx40 GJ plaques following hypoxia (see Figure 2). Surprisingly, despite the presence of GJ plaques in Jeg/hCx40 cells, they remained uncoupled in normoxia. However, after 24 h of hypoxia, the presence of DAPI transfer between cell patches became evident, resembling the patches of Cx40 GJ plaques observed by immunofluorescence (Figure 3, right panel).

### 3.3. Cx40 Modulates Hypoxia-Induced Changes in Migration and Proliferation of Jeg-3 Cells

**Figure 4:**
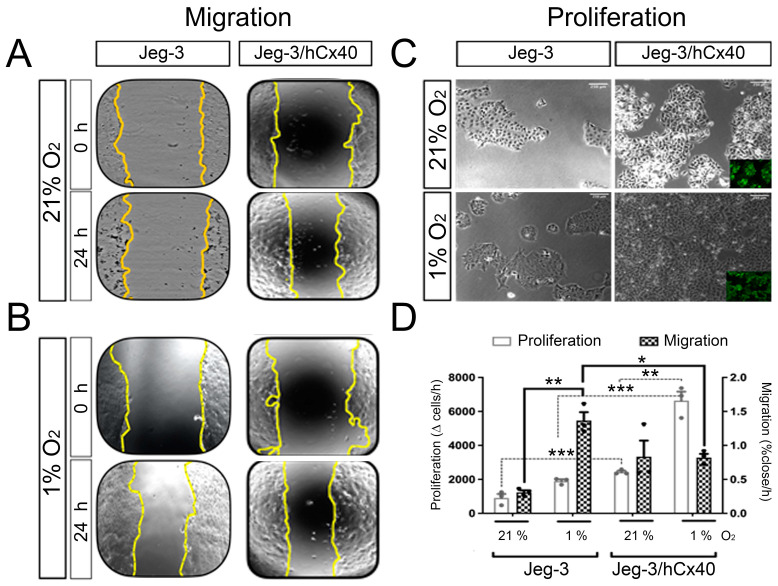
Cx40 levels modulate the effect of hypoxia in Jeg-3 cells. Jeg-3 and Jeg- 3/hCx40 cells were grown in hypoxia for 24 h and compared to controls (normoxia). Migration was evaluated by wound healing test. Yellow line draws the migration front: (**A**) upper panels show cells maintained in normoxia or (**B**) lower panels in hypoxia. (**C**) Proliferation assay, upper images correspond to cells grown in normoxia, and lower images correspond to cells grown in hypoxia. The photographs correspond to the cell density at 24 h. Inserts show GFP expression of Jeg-3/hCx40 cells. (**D**) Quantification of proliferation and migration (left and right abscissa respectively). The graph shows the average rate of change for each condition. Statistical comparison by ANOVA: * *p* < 0.05, ** *p* < 0.01; and *** *p* < 0.005; bars represent the standard error (n = 3). Only comparisons whose *p* was <0.05 are drawn, any other multiple comparison not indicated in the graph was not significant.

As mentioned previously, both hypoxia and Cx40 expression are linked to changes in trophoblast proliferation and migration. Therefore, we assessed whether hypoxia and Cx40 could interact, potentially producing a synergic outcome. Regarding migration, as determined by the wound healing assay, Jeg-3 cells under normoxia exhibited a low closure velocity (Figure 4A–D, 0.31 ± 0.04% closure/h). However, they migrated significantly faster when cultured under hypoxic conditions (Figure 4B–D, 1.35 ± 0.14% closure/h). On the other hand, 24 h of hypoxia induced a tendency to increase the proliferation rate of Jeg-3 cells, but this was not statistically significant (Figure 4C,D). We considered a possible explanation for this observation, which could be that an increase in cell proliferation balances an increase in cell death. To address this possibility, we investigated cell viability by assessing the uptake of a commonly used fluorescence molecule, 7-AAD, known for its role in determining cell death [31,32]. Our findings did not reveal any evidence of cell death, both in the control condition and after 24 h of hypoxia. Collectively, these results suggest that the observed increase in migration cannot likely be attributed to an increase in the number of cells. Interestingly, under normoxic conditions, the Jeg-3/hCx40 cells demonstrated a higher migration capacity compared to the Jeg-3 cells. However, hypoxia did not enhance the migration capacity in the Jeg-3/hCx40 cells (Figure 4B–D, 0.82 ± 0.23 vs. 0.80 ± 0.07% closure/h). Moreover, in contrast to the observed effect in the Jeg-3 cells, hypoxia increased the proliferative rate in cells with a high Cx40 expression (Figure 4C,D). These findings suggest that, under hypoxic conditions, cells with low Cx40 levels exhibit a more “migratory” phenotype, whereas hypoxia in cells with high levels of Cx40 promotes a more “proliferative” phenotype. Thus, it appears that Cx40 levels are a crucial factor in determining the final outcome induced by hypoxia.

### 3.4. NO Enhanced Cx40 GJ Plaques, but Reduced Migration and Proliferation of Jeg-3 Cells

**Figure 5:**
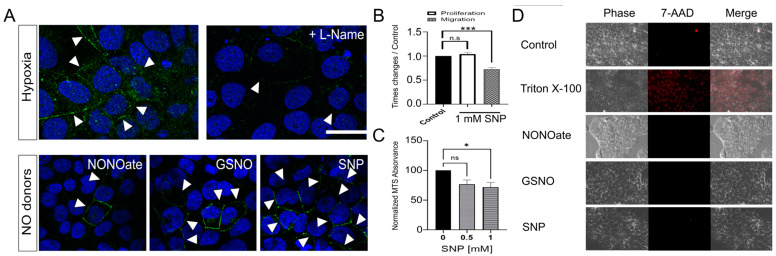
NO enhanced Cx40 GJC, but reduced migration and proliferation of Jeg-3 cells. Jeg-3 cells were maintained in normoxia or hypoxia alone or in combination with NO modulators for 24 h (**A**) Jeg-3 cells were grown in culture media with a nitric oxide synthase inhibitor (L-NAME) or in presence of NO donors (1 mM SNP, 500 µM GSNO, or 500 µM NONOate) for 24 h and then the localization of Cx40 (green signal) was studied by immunofluorescence in a confocal microscope. Nuclei were stained with DAPI and arrow heads show localization of Cx40 GJ-plaques. (**B**) Graphic shows the effect of 1 mM SNP on the proliferation and migration capacity of Jeg-3 cells. Statistical comparison by ANOVA: *** *p* <0.001, ns *p* >0.05 (n = 4). (**C**) Jeg-3 cells were cultured in media containing 0.5- and 1-mM SNP for 24 h, and cell viability was subsequently assessed using an MTS kit. (**D**) Jeg-3 cells were cultured in media with various NO donors (1 mM SNP, 500 μM GSNO, or 500 μM NONOate) for 24 h, and the uptake of 10 μM 7-AAD was measured. As a positive control for this technique, cells were exposed to culture media with 0.5% Triton X-100. In all cases, after 10 min of exposure to 7-AAD, cells were washed and images were captured using a microscope. The scale bars = 50 μm (n = 3). Statistical comparison by ANOVA: ns > 0.05; * *p* < 0.05, and *** *p* < 0.005; bars represent the standard error (n = 3).

As demonstrated, hypoxia induced changes in the Cx40 distribution in the Jeg-3 cells, correlating with alterations in the migration and proliferation of these cells. It is well known that hypoxia can elevate NO production in trophoblast cells [33]. In the placenta, NO is implicated in EVTs’ migration and is also associated with the pathogenesis of preeclampsia [34,35]. Therefore, we investigated whether NO could mimic the effects of hypoxia in Jeg-3 cells concerning the Cx40 localization, migration, and proliferation profile. Firstly, we assessed the impact of 1 mM L-Name, a nitric oxide synthase inhibitor, on the formation of Cx40 GJ plaques in Jeg-3 cells exposed to 24 h of hypoxia. As previously demonstrated, hypoxic conditions led to an increase in both the number and thickness of Cx40 GJ plaques in the Jeg-3 cells (Figure 5A, left upper panel). However, when cells were subjected to hypoxia in the presence of L-Name, GJ plaques were nearly absent, or when present, they were thin (Figure 5A, right upper panel). Next, we explored whether NO played a role in GJ plaque formation, hypothesizing that different NO donors could mimic the effects of hypoxia. Jeg-3 cells were exposed to 1 mM of the NO donor Sodium Nitroprusside (SNP), 500 µM of S-nitrosoglutathione (GSNO), or 500 µM of NONOate for 24 h. All three NO donors induced an increase in GJ plaque formation, although with some variations. NONOate was the least effective in terms of the frequency of GJ plaques observed in a given field, but the plaques were still noticeable (Figure 5A, lower panels). In the case of GSNO, it increased both the frequency and thickness of GJ plaques. SNP, on the other hand, boosted the number of GJ plaques between cells. Subsequently, we investigated the impact of SNP, the most effective GJ plaque inducer, on Jeg-3 cell proliferation and migration. Surprisingly, exposure to 1 mM SNP for 24 h did not reduce proliferation, but significantly hindered the migration capacity of Jeg-3 cells (Figure 5B).

Given that high concentrations of NO can potentially cause cell damage [36], which may account for its lack of effect on Jeg-3 cell division, we conducted tests to assess whether 1 mM SNP could induce cell death. After 24 h of exposure to 0.5- or 1-mM SNP, we observed ~24% and ~29% decreases in MTS-associated absorbance, respectively (Figure 5C). This suggests that SNP at these concentrations reduces cell viability. However, under phase-contrast microscopy, we did not observe any noticeable cell damage. Accordingly, under normal conditions, we found no evidence of 7-AAD uptake. However, after cell permeabilization with 0.5% Triton X-100, the cell nuclei became denser, some blebs were observed, and 100% of the cells showed uptake of 7-AAD (Figure 5D). Importantly, the exposure of Jeg-3 cells to 500 µM NONOate, 500 µM GSNO, or 1 mM SNP did not induce 7-AAD uptake in any of the cells. These results suggest that 1 mM SNP induces certain changes in mitochondrial function, as indicated by the MTS signal’s strength, which is linked to mitochondrial redox capacity. However, these cellular modifications were not substantial enough to induce cell death.

### 3.5. GMPc/PKG Induces Cx40 GJ Plaques between Jeg-3 Cells

**Figure 6:**
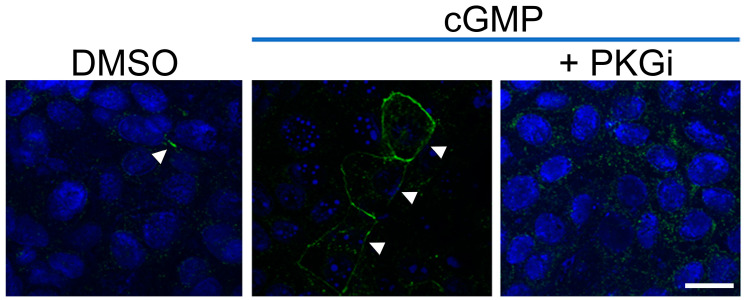
cGMP/PKG induces Cx40 GJ plaques between Jeg-3 cells. Jeg-3 cells were cultured in 21% O_2_ and treated with DMSO (0.1%) (**left panel**), 1 mM 8-bromo-cGMP (**middle panel**), or 1 mM 8-bromo-cGMP plus 1 μM PKGi (KT5823, **right panel**). Arrow heads denote the presence of GJ plaques between cells. Pictures representatives of 3 independent experiments. Scale bar = 20 µm.

One of the primary pathways activated by NO is the cGMP/PKG-dependent pathway, which appears to play a role in trophoblast migration and invasion [37]. Additionally, both NO and cGMP have been shown to modulate Cx40 GJC-mediated communication in HeLa cells [38]. Therefore, we investigated whether a cGMP/PKG-dependent pathway is involved in Cx40 GJ plaque formation in Jeg-3 cells. In the control condition, where Jeg-3 cells were treated for 24 h with only DMSO (the vehicle for 8-bromo-cGMP, a cGMP donor), the Cx40 signal was primarily observed in the cytoplasm (Figure 6, upper panel). However, when treated with 1 mM cGMP, a portion of the protein translocated to the plasma membrane, forming GJ plaques (Figure 6, middle panel, white arrow), as observed in hypoxia (Figure 2) and with a NO donor (Figure 5). Nevertheless, when cells were treated with cGMP and a PKGi, the frequency of plaques in the cell membrane decreased, and they also became thinner (Figure 6, lower panel, white arrowheads). These results suggest that the cGMP/PKG pathway may be involved in hypoxia/NO-induced Cx40 GJ plaque formation in Jeg-3 cells.

## 4. Discussion

**Figure 7:**
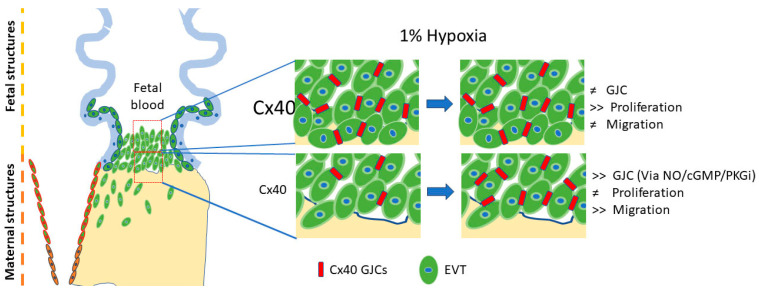
Model of the relationship between Cx40 levels and the effect of hypoxia on EVTs. As hypoxia is established, it will increase proliferation in cells with high Cx40 expression levels and migration in cells with low Cx40 expression levels. In addition, hypoxia-induced Cx40 GJ plaques formation in cells with low Cx40 levels possibly occurs via a NO/cGMP/PKG-dependent pathway. However, cells with high expression basally form Cx40 GJCs, which seem to not be affected by hypoxia.

In this study, we discovered that hypoxia enhances the formation of Cx40 GJ plaques between neighboring cells, a process hindered by a nitric oxide synthase inhibitor (L-name). Consistent with this finding, exposure to NO donors (NONOate, GSNO, and SNP), along with a cGMP donor, also augmented GJ plaque formation. The addition of a PKG inhibitor reduced cGMP-induced Cx40 GJ plaque formation. On the contrary, hypoxia increased Jeg-3 cell migration, a phenomenon prevented in cells overexpressing Cx40 during hypoxia. Unexpectedly, treatment with 1 mM SNP did not alter the proliferation rate of Jeg-3 cells; however, it significantly reduced their migration capacity. These results suggest that Cx40 induces a shift in the hypoxia-induced response in Jeg-3 cells. Furthermore, it seems that hypoxia induces an increase in Cx40 GJ plaques, a response mimicked by the activation of the NO/cGMP-PKG pathway, suggesting a potential interplay between these molecular mechanisms. However, the impact of hypoxia on migration and proliferation appears to be more intricate and does not necessarily require a NO-dependent mechanism. Finally, these findings shed light on the effects of hypoxia on EVTs located close to fetal structures (expressing high Cx40 levels) compared to those close to maternal structures (expressing low or very low Cx40 levels), hinting at a potential involvement of Cx40 in the generation or worsening of preeclampsia (Figure 7).

Anchoring villi form columns where three main cell types can be identified: proliferative EVTs, which, as the name implies, constitute a subpopulation with proliferative capacity; immature or intermediate EVTs, maintaining proliferative properties but exhibiting a greater migratory capacity without the ability to invade tissues; and invasive/migratory EVTs, which lose their proliferative properties while acquiring enhanced migratory and invasive abilities [4,5,35]. In a prior study by Nishimura and colleagues, blocking Cx channels with carbenoxolone (CBX) correlated with a reduction in proliferation, but an enhancement of EVTs’ migration [28]. Additionally, Genbacev [11] demonstrated that hypoxia acts as a stimulus for EVTs’ migration. Therefore, it seems logical to consider that hypoxia, at least in part, acts by inhibiting Cx40 GJC function. However, in the present study, we observed that hypoxia promoted the formation of Cx40 GJ plaques at cell–cell contacts. Discrepancies between our findings and those reported in prior studies could be attributed, in part, to differences in the stimuli evaluated and the specific cell types affected by these stimuli. Nishimura evaluated the effect of abolishing Cx40 function on placental explants grown at 3% O_2_. Despite its proximity to real-life conditions, this model has the disadvantage of not distinguishing the effects on proliferative EVTs, immature EVTs, and migratory EVTs, or a combination of them. However, based on our results, the impact of hypoxia might vary depending on the levels of Cx40 expressed. The overexpression of Cx40 in Jeg-3 cells (Jeg-3/hCx40), akin to proliferative EVTs, restricts the migratory effect of hypoxia. Conversely, Jeg-3 cells expressing lower quantities of Cx40, resembling an intermediate (immature) EVT population, are sensitive to hypoxia, promoting increased migration. This implies that Cx40 expression levels could act as an ‘on–off switch’ for the migratory effect of hypoxia, segregating these two cell subpopulations and representing a regulatory brake for cell migration, independent of the migratory stimulus. Similar results were reported by Wright et al., where the overexpression of Cx40 in Jeg-3 cells was associated with a decrease in cell migration induced by EGF [39].

A dominant assumption regarding cells connected through GJCs is that they are tightly ‘docked’ to each other, implying limited mobility. Contrary to this notion, our study revealed a positive correlation between increased Cx40 GJ plaque formation and an enhanced cell migration capacity. It is essential to note that GJ plaques are dynamic structures with reported half-lives ranging from 1 to 5 h [40,41]. Our migration experiments spanned 24 h, allowing ample time to observe multiple cycles of GJC degradation, cell migration, and subsequent GJ plaque re-formation. However, we did not explore the dynamic behavior of Cx40 GJ plaques over time, and this hypothesis warrants testing in future experiments. Moreover, it is conceivable that Cx40 might influence cell migration independently of GJC function. For instance, Cx43 has been demonstrated to enhance human umbilical vein endothelial cell (HUVEC) migration by interacting with SHP-2 [42], a phosphatase known to regulate cell motility [43]. Additionally, Cx43 has been implicated in controlling astrocytic motility through hemichannels that release signaling molecules [44,45]. In the case of Cx40, prior studies have indicated that HeLa cells expressing Cx40 migrate faster than those expressing other types of Cxs. This heightened migration has been attributed to the formation of nanotubes and the transfer of small interfering RNAs (siRNAs), suggesting a potential role for Cx40 in cell migration [46].

As illustrated in Figure 3, differences are evident in cell proliferation and migration when comparing Jeg-3 cells with low and high expressions of Cx40 under both normoxic and hypoxic conditions. While it is reasonable to assume that cells with a higher proliferation rate might close a wound or gap more quickly, not necessarily due to an enhanced migratory capacity, but simply because there are more cells to participate in the process, our findings in this study reveal an intriguing contrast. Jeg-3/hCx40 cells exhibited a higher proliferation rate under hypoxic conditions, yet their migration rate remained relatively consistent when comparing oxygen levels at 21% and 1%. This suggests that the influence of hypoxia on migration in Jeg-3/Cx40 cells could be even more pronounced. In contrast, Jeg-3 cells exposed to hypoxia showed an approximately two-fold increase in their proliferation rate. Notably, their migration rate increased by about six-fold. This substantial disparity emphasizes that, irrespective of the variations in cell proliferation in Jeg-3 cells, hypoxia itself exerts a significant impact on the migration of these cells.

It is well known that NO can activate cGMP- and PKG-dependent pathways in several cell types [47,48,49], which seems to be involved in both trophoblast migration and invasion [37]; moreover, both NO and cGMP modulate Cx40 GJ-mediated communication in Hela cells [38]. In this work, we observed an increase in the translocation of Cx40 to the cell membrane when using cGMP, which was decreased in the presence of a PKG inhibitor, suggesting that both hypoxia and the activation of a NO/cGMP/PKG dependent pathway are capable of triggering a change in Cx40 cell location. Accordingly, an increase in cell communication mediated by GJC and an increase in cell migration have been observed in certain cancer cells [50,51] and in migratory neural crest cells [52]. However, as mentioned before, the blocking of connexin channels with carbenoxolone (CBX) is correlated with the promotion of EVTs’ migration [28]. But, it has been reported that CBX, despite an initial reduction in intercellular communication, after 6 h induced an increase in Cx43 expression and Cx43-GJ plaque formation in PKA-dependent pathways in bovine aortic endothelial cells [53]. Also, CBX can inhibit Cx43 hemichannels, as well other ion channels in astrocytes [54]. Thus, CBX-mediated results should be interpreted carefully, and it may be that the relationship between Cx40, GJCs’ formation and function (and hemichannels as well), and trophoblast migration is more complex than originally suspected.

Finally, despite Jeg-3 cells being extensively used as a model for trophoblast studies, we recognize that other cell models (e.g., HTR8, Swan71, and SGHPL4/5) may be more physiologically relevant. Therefore, we encourage other research groups to replicate our experiments to verify whether our results truly reflect the dynamics of the potential crosstalk between Cx40 expression and hypoxia in the placenta.

## Data Availability

All results will be available as requested.
